# The causal association between maternal smoking around birth on childhood asthma: A Mendelian randomization study

**DOI:** 10.3389/fpubh.2022.1059195

**Published:** 2022-11-03

**Authors:** Zijun Ding, Lei Pang, Hongqiang Chai, Fei Li, Ming Wu

**Affiliations:** ^1^Department of Neonatology, Shanxi Children's Hospital, Taiyuan, China; ^2^Department of Urology, The Fifth Hospital of Shanxi Medical University (Shanxi Provincial People's Hospital), Taiyuan, China

**Keywords:** maternal smoking around birth, childhood asthma, maternal Alzheimer's disease, maternal hypertension, maternal heart disease, Mendelian randomization

## Abstract

To explore the causal relationship between maternal smoking around birth and childhood asthma using Mendelian randomization (MR). Using the data from large-scale genome-wide association studies, we selected independent genetic loci closely related to maternal smoking around birth and maternal diseases as instrumental variables and used MR methods. In this study, we considered the inverse variance weighted method (MR-IVW), weighted median method, and MR-Egger regression. We investigated the causal relationship between maternal smoking around birth and maternal diseases in childhood asthma using the odds ratio (OR) as an evaluation index. Multivariable MR (MVMR) included maternal history of Alzheimer's disease, illnesses of the mother: high blood pressure and illnesses of the mother: heart diseaseas covariates to address potential confounding. Sensitivity analyses were evaluated for weak instrument bias and pleiotropic effects. It was shown with the MR-IVW results that maternal smoking around birth increased the risk of childhood asthma by 1.5% (OR = 1.0150, 95% CI: 1.0018–1.0283). After the multivariable MR method was used to correct for relevant covariates, the association effect between maternal smoking around birth and childhood asthma was still statistically significant (*P* < 0.05). Maternal smoking around birth increases the risk of childhood asthma.

## Introduction

Maternal smoking has many adverse effects on fetal and child health. Despite various smoking cessation measures, about 11% of women smoke during pregnancy, exposing the fetus in their uterus to smoke (1). It has been found in previous studies that the occurrence and development of childhood asthma were associated with maternal smoking during pregnancy. Still, their perspective is limited to traditional observational epidemiology, which is susceptible to unknown confounding and reverse causality (2). In recent years, Mendelian randomization (MR) has been developed as a crucial causal inference method. Genetic variation is used in this method as an instrumental variable, allowing the defects of traditional epidemiological studies, such as difficulty in data acquisition and poor extrapolation of results (3), to be overcome. Two sample and multivariate MR methods were used in this study to analyze aggregated data from the genome-wide association study (GWAS) to investigate the causal association between maternal smoking and childhood asthma.

## Materials and methods

### Data sources

Maternal smoking around birth was used as the exposure variable in this study. The outcome variable was childhood asthma. A covariate was used in the multivariate MR maternal history of Alzheimer's disease, illnesses of the mother: high blood pressure, illnesses of the mother: heart disease, all the data from the website (https://gwas.mrcieu.ac.uk/datasets). The above GWAS data are from the European origin population, and their brief information is shown in Table 1.

**Table 1:** Brief description of the genome-wide association study data used in this study.

**GWAS ID**	**Year**	**Trait**	**Consortium**	**Sample size**	**Number of SNPs**
ukb-b-17685	2018	Maternal smoking around birth	MRC-IEU	397,732	9,851,867
ebi-a-GCST005923	2018	Maternal history of Alzheimer's disease	NA	288,676	7,776,415
ukb-b-18167	2018	Illnesses of the mother: high blood pressure	MRC-IEU	426,391	9,851,867
ukb-b-12477	2018	Illnesses of the mother: heart disease	MRC-IEU	426,240	9,851,867
ukb-d-ASTHMA_CHILD	2018	Childhood asthma (age <16)	NA	361,194	10,443,939

### Data collation

Independent single nucleotide polymorphisms (SNPs) with genome-wide significance associated with maternal smoking around birth and maternal diseases were selected from the database as instrumental variables. This procedure was performed to avoid bias caused by strong linkage disequilibrium (LD) among the SNPs in the analysis. The screening criteria were as follows: ① the genome-wide significance of maternal diseases and living habits was determined based on the genome-wide information of the 1,000 Genomes Project (*P* < 5 × 10^−8^), ② the physical distance between every two genes >1,0000 kb, and ③ *R*^2^ threshold of LD between genes was < 0.01 (4). See Table 2 for instrumental variables.

**Table 2:** Basic information on SNPs associated with maternal smoking around birth and maternal diseases.

**SNP**	**Effect allele**	**Other allele**	**Beta**	**Eaf**	**Se**	**Pval**
rs10226228	G	A	0.0074	0.3702	0.0011	4.00E-12
rs12405972	T	G	−0.0081	0.3483	0.0011	6.40E-14
rs12923476	A	G	−0.0069	0.2569	0.0012	4.80E-09
rs1323341	G	A	−0.0068	0.7816	0.0012	3.90E-08
rs2183947	A	G	−0.0078	0.2250	0.0012	1.50E-10
rs2428019	A	C	0.0070	0.2393	0.0012	5.10E-09
rs35566160	G	A	0.0061	0.2748	0.0012	4.50E-08
rs36072649	A	T	−0.0072	0.3808	0.0011	1.10E-11
rs4865667	T	C	−0.0058	0.3878	0.0011	3.40E-08
rs576982	T	C	−0.0093	0.2279	0.0012	2.30E-14
rs6011779	T	C	−0.0099	0.8087	0.0013	2.50E-14
rs62477310	C	T	−0.0058	0.4867	0.0010	2.00E-08
rs7002049	C	T	0.0076	0.7847	0.0012	1.40E-09
rs75596189	T	C	0.0121	0.1100	0.0016	2.10E-13
rs7899608	T	C	0.0088	0.1413	0.0015	2.30E-09
rs794356	A	G	−0.0061	0.4421	0.0011	1.90E-08
rs2965169	C	A	−0.1056	NA	0.0125	3.40E-17
rs429358	C	T	0.5927	NA	0.0113	1.00E-200
rs56394238	G	A	0.1352	NA	0.0123	4.12E-28
rs6733839	T	C	0.1156	NA	0.01226	4.00E-21
rs10061288	G	A	−0.0058	0.5149	0.0010	5.30E-09
rs10876539	G	A	−0.0058	0.4954	0.0010	7.80E-09
rs11191515	A	G	−0.0104	0.0775	0.0019	2.20E-08
rs11642572	T	C	0.0059	0.5963	0.0010	6.20E-09
rs117913411	A	T	0.0176	0.0343	0.0028	2.40E-10
rs12143461	A	G	−0.0060	0.3262	0.0011	1.90E-08
rs12188031	C	T	−0.0059	0.3616	0.0010	1.50E-08
rs12509595	C	T	0.0079	0.2917	0.0011	4.70E-13
rs1275984	C	A	−0.0085	0.6162	0.0010	1.20E-16
rs12978472	G	C	−0.0091	0.1293	0.0015	9.70E-10
rs17473410	G	T	0.0101	0.0791	0.0019	4.60E-08
rs1887320	A	G	0.0063	0.4754	0.0010	3.20E-10
rs1894400	T	C	0.0105	0.3253	0.0011	6.40E-23
rs1950764	A	G	−0.0076	0.1719	0.0013	7.70E-09
rs2210601	T	C	0.0087	0.3858	0.0010	1.30E-17
rs2478539	T	G	0.0063	0.4031	0.0010	4.80E-10
rs347617	T	C	0.0058	0.6684	0.0011	3.50E-08
rs35427	G	T	−0.0074	0.3828	0.0010	2.00E-12
rs3753584	C	T	−0.0079	0.1623	0.0014	4.10E-09
rs3918226	T	C	0.0104	0.0810	0.0019	1.90E-08
rs41849	C	T	0.0060	0.3404	0.0010	1.10E-08
rs429358	C	T	−0.0104	0.1541	0.0014	5.10E-14
rs4917676	T	C	0.0086	0.8318	0.0013	1.60E-10
rs592373	A	G	0.0070	0.6297	0.0010	1.10E-11
rs602543	T	C	−0.0060	0.3957	0.0010	3.70E-09
rs6026742	A	G	0.0091	0.1177	0.0016	4.00E-09
rs633185	C	G	0.0066	0.7151	0.0011	2.80E-09
rs72831345	A	G	−0.0077	0.1452	0.0014	4.50E-08
rs764124	A	G	−0.0069	0.2448	0.0012	3.10E-09
rs1047891	A	C	−0.0054	0.3159	0.0010	6.30E-09
rs117733303	G	A	0.0371	0.0184	0.0032	8.60E-31
rs2071475	A	G	0.0060	0.2090	0.0011	1.90E-08
rs4299376	T	G	−0.0052	0.6769	0.0010	2.30E-08
rs72807674	C	A	0.0054	0.3002	0.0010	1.20E-08
rs74617384	T	A	0.0210	0.0789	0.0016	4.30E-39
rs8043119	A	G	−0.0052	0.4277	0.0009	4.40E-09
rs9349379	G	A	0.0050	0.4056	0.0009	1.80E-08

### Statistical treatment

#### Main analysis method

Inverse variance weighted (MR-IVW) was used as the primary analysis method to evaluate the causal effects of maternal smoking around birth and maternal diseases on childhood asthma. The MR-IVW application assumes that all SNPs are valid instrumental variables. The calculation formula is as follows:


(1) $$\begin{array}{l} {\hat{B}}_{MR-IVW}=\frac{{\underset{i}{\sum }}^{w_{i}}{\hat{B}}_{j}}{\overset{w_{j}}{\underset{i}{\sum }}},w_{j}=\frac{{{\hat{\gamma }}_{j}}^{2}}{\sigma Yj^{2}} \end{array}$$

Where σYj2 is the variance of the estimated gene-outcome association value of the JTH instrumental variable. The inverse variance weighting method uses the weight Wj to weight the Wald ratio of the instrumental variable to calculate the degree of association between exposure and outcome.

#### Sensitivity analysis

MR analysis is a powerful tool in epidemiological studies. In this study, the inverse variance weighted (IVW), MR-Egger regression, and weighted median estimator (WME) were used for MR analysis. The IVW principle takes the inverse of the variance of each instrumental variable as the weight for weighted calculation on the premise that all instrumental variables are valid. The intercept term is not considered in regression, and the final result is the weighted average of the effect values of all instrumental variables (5). The most significant difference between the MR-Egger method and IVW is that the existence of an intercept term is considered in regression. Additionally, it uses the inverse of outcome variance as the weight for fitting. WME is the median of the weighted empirical density function, defined as the estimated value of the ratio. If there are at least half the valid tools in the analysis, causality can be consistently estimated (6).

#### Multivariate Mendelian randomization method

Multivariate MR methods were used to adjust for covariates: maternal history of Alzheimer's disease, illnesses of the mother: high blood pressure, illnesses of the mother: heart disease. SNPs used as instrumental variables in multivariable MR should meet the following conditions: ① SNPs are associated with all exposure factors in the model, ② they do not affect the outcome variables in other ways, and ③ the number of SNPs is greater than the number of exposure factors (7).

The above analysis was performed in R v.4.1.2. MR-IVW, MR-WME, and MR-Egger were made with the R packages “Two Sample MR” and MR-PRESSO. Multivariable MR methods were made with the R packages MR-PRESSO and Mendelian Randomization (7), respectively. The evaluation indexes were odds ratio (OR) and 95% confidence interval (CI). *P* < 0.05 was considered statistically significant (two-sided).

## Results

### Results of univariate Mendelian randomization

**Figure 1:**
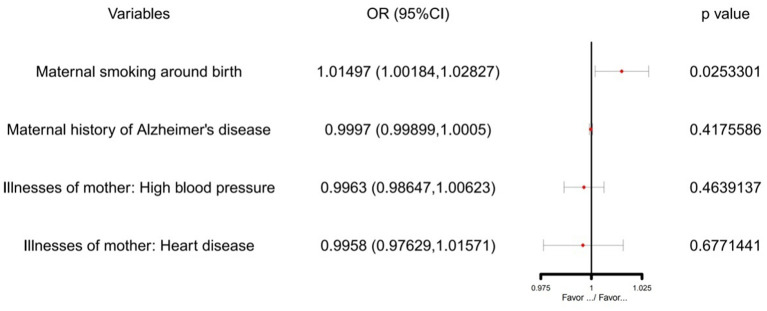
The SVMR forest plot of maternal smoking around birth on childhood asthma susceptibility using SNPs.

According to the MR-IVW test results, maternal smoking around birth was associated with the risk of childhood asthma (*P* < 0.05). In contrast, maternal history of Alzheimer's disease, illnesses of the mother: high blood pressure, and illnesses of the mother: heart disease were not statistically significant in MR-IVW test results (*P* > 0.05) (see Figure 1).

IVW showed that maternal smoking around birth (OR = 1.014970, 95% CI: 1.0018377–1.028274) was associated with an increased risk of childhood asthma (Table 3). However, there was no evidence of the association between maternal history of Alzheimer's disease (IVW OR = 0.9997026, 95% CI: 0.9989838–1.000422), Illnesses of the mother: high blood pressure (IVW OR = 0.9963000, 95% CI: 0.9864658–1.006232), Illnesses of the mother: heart disease (IVW OR = 0.9958047, 95% CI: 0.9762907–1.015709) and childhood asthma. Similar patterns of association were observed in the weighted median and MR-Egger regression methods.

**Table 3:** Causal effects of four types of maternal smoking around birth and maternal diseases on childhood asthma based on two-sample Mendelian randomization.

**SNPs\Methods**	**MR-egger**	**Weighted median**	**IVW**
Maternal smoking around birth	0.470 (−0.043 to 0.096)	0.143 (−0.005 to 0.031)	0.025 (0.002 to 0.028)
Maternal history of Alzheimer's disease	0.817 (−0.001 to 0.001)	0.509 (−0.001 to 0.001)	0.418 (−0.001 to 0.000)
Illnesses of the mother: high blood pressure	0.647 (−0.054 to 0.034)	0.807 (−0.015 to 0.012)	0.464 (−0.014 to 0.006)
Illnesses of the mother: heart disease	0.297 (−0.012 to 0.046)	0.923 (−0.022 to 0.024)	0.677 (−0.024 to 0.016)

The Cochran *Q*-test of IVW showed that there was no heterogeneity (maternal smoking around birth: *Q* = 13.020, *P* = 0.601; maternal history of Alzheimer's disease: *Q* = 1.821, *P* = 0.610; Illnesses of the mother: high blood pressure: *Q* = 22.065, *P* = 0.778; Illnesses of the mother: heart disease: *Q* = 8.875, *P* = 0.262). Besides, we did not detect any evidence of horizontal pleiotropy based on the intercept analysis of Egger regression (maternal smoking around birth: intercept = −8.67E-05, *P* = 0.748629; maternal history of Alzheimer's disease: intercept = −5.30E to 05, *P* = 0.745886; Illnesses of the mother: high blood pressure: intercept = 5.18E−05, *P* = 0.761886; Illnesses of the mother: heart disease: intercept = −0.000219009, *P* = 0.123426).

The “leave-one-out” sensitivity analysis results showed that after removing each SNP in turn, the IVW analysis results of the remaining SNPs were close to the analysis results of the inclusion of all SNPs, suggesting no SNPs had a strong impact on the estimated causal association.

### Results of multivariate Mendelian randomization

**Figure 2:**
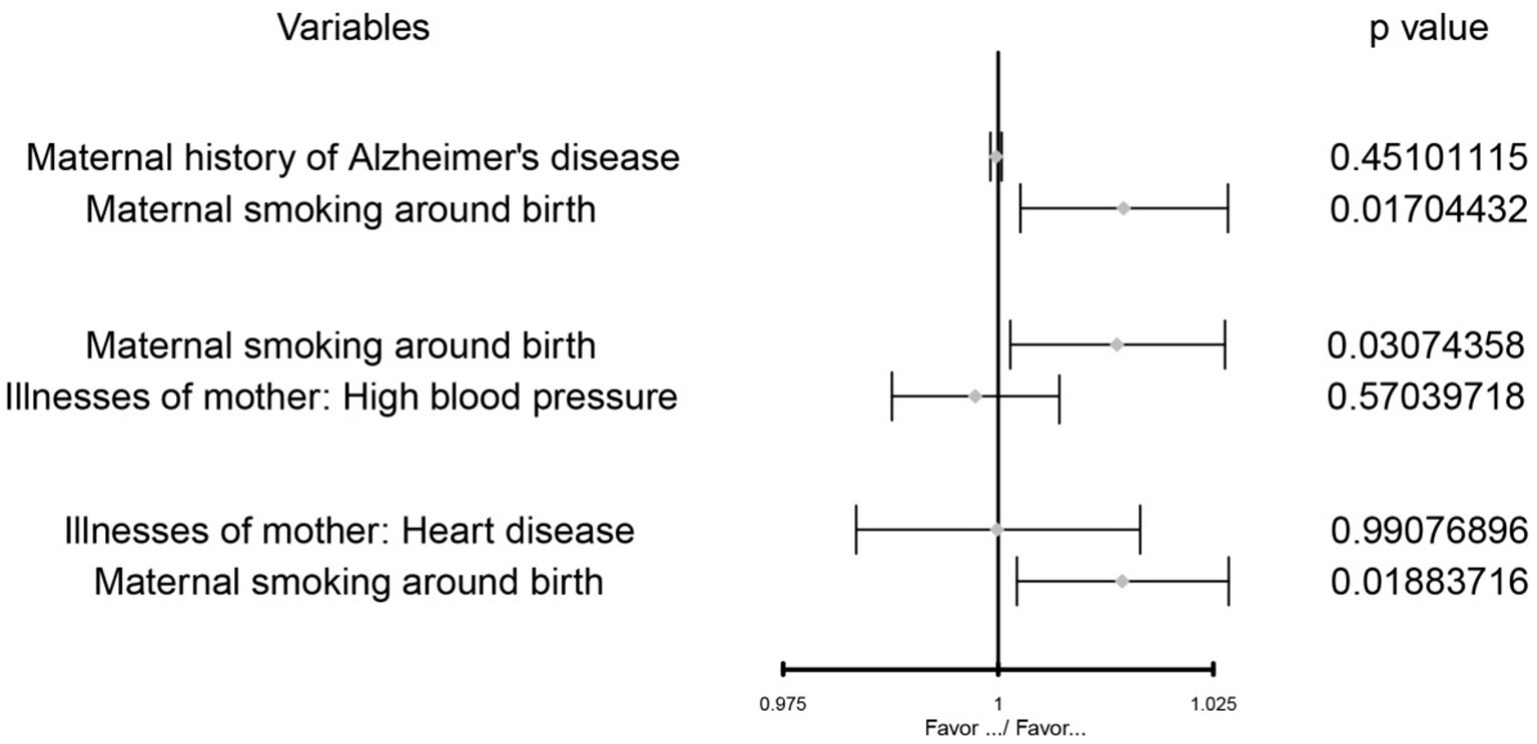
The MVMR forest plot of maternal smoking around birth on the maternal history of Alzheimer's disease, illnesses of the mother: high blood pressure, illnesses of the mother: heart disease separately.

**Figure 3:**
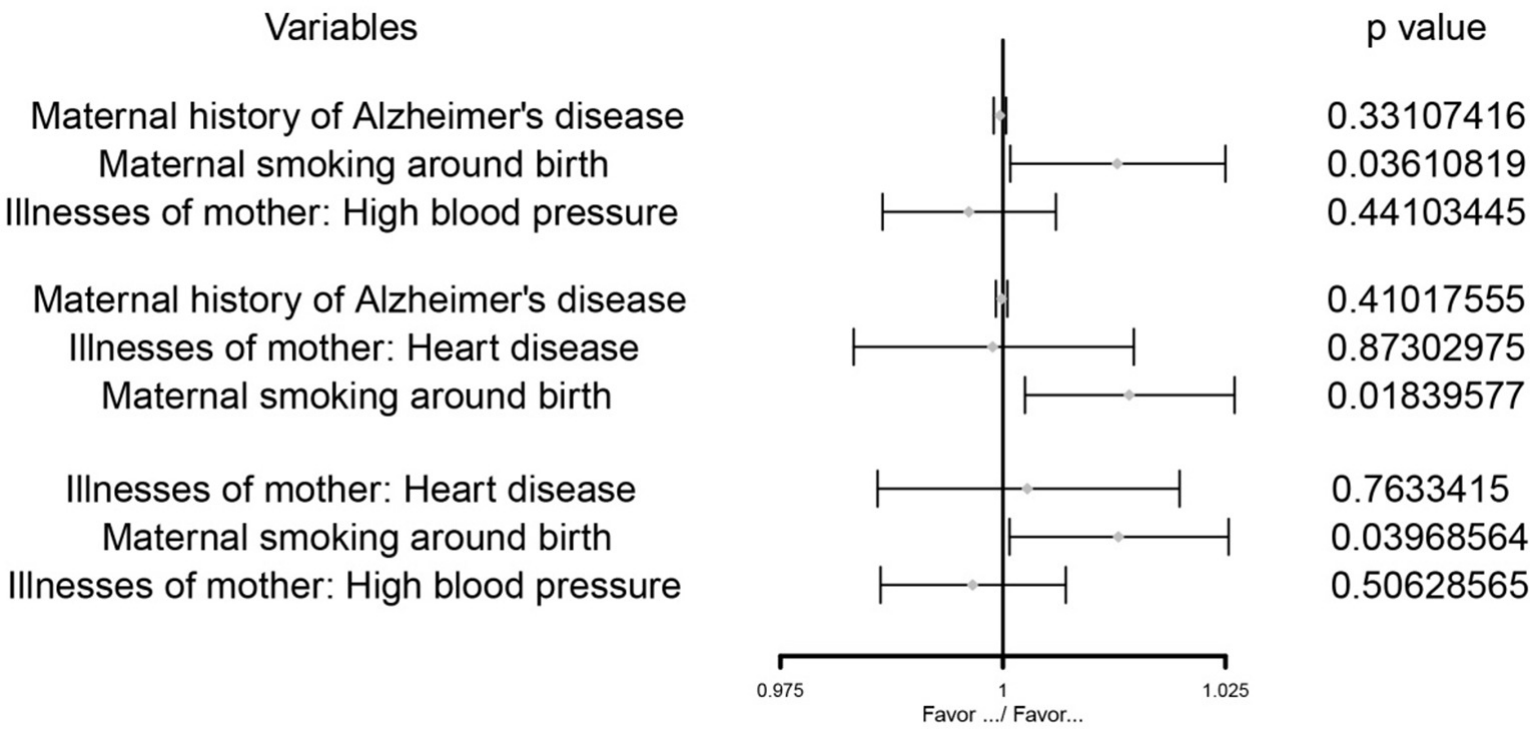
The MVMR forest plot of maternal smoking around birth on childhood asthma controlling the maternal history of Alzheimer's disease and illnesses of the mother: high blood pressure, the maternal history of Alzheimer's disease and illnesses of the mother: heart disease, illnesses of the mother: high blood pressure and illnesses of the mother: heart disease separately.

**Figure 4:**
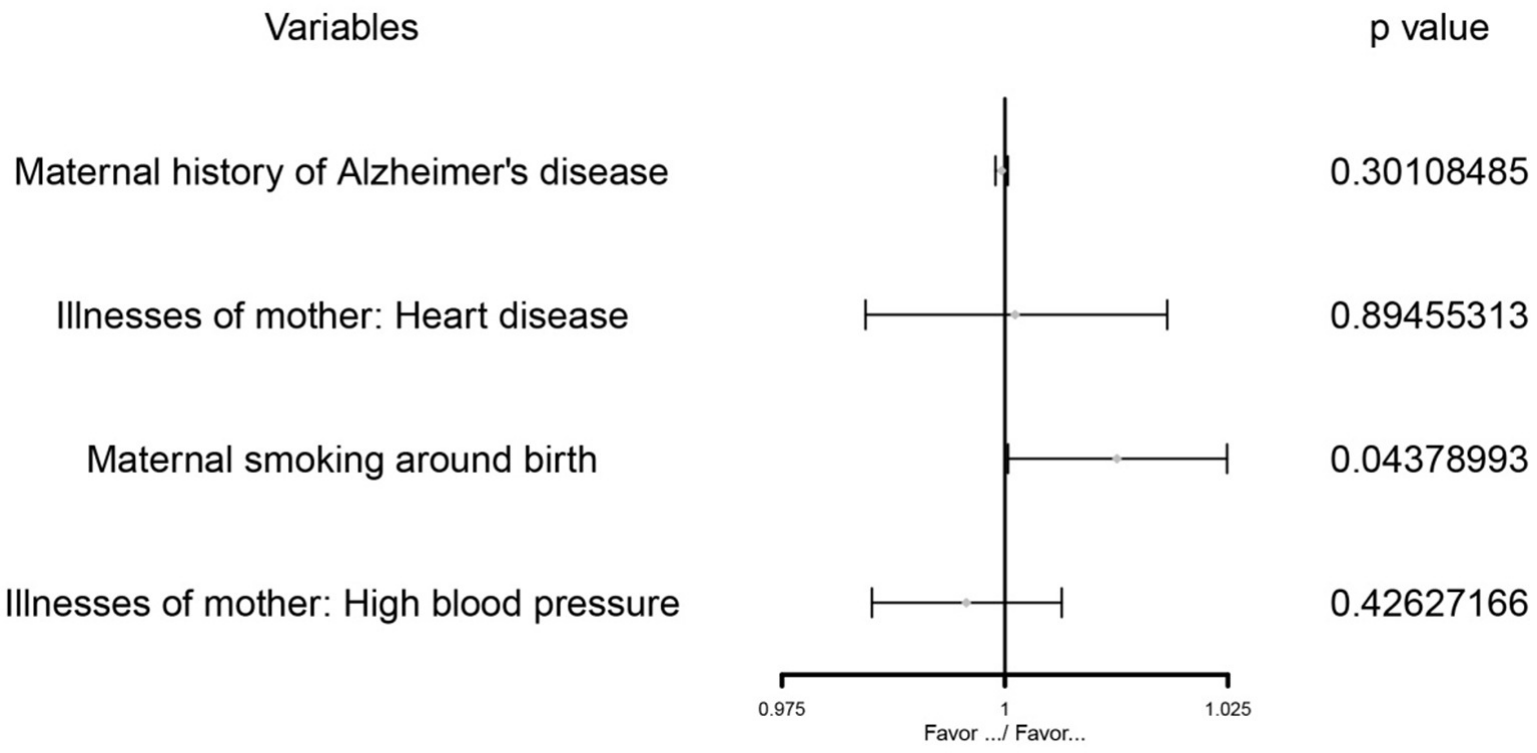
The MVMR forest plot of maternal smoking around birth on childhood asthma controls the maternal history of Alzheimer's disease, illnesses of the mother: high blood pressure, and illnesses of the mother: heart disease.

After multivariate MR adjustment for covariates (maternal history of Alzheimer's disease, illnesses of the mother: high blood pressure, and illnesses of the mother: heart disease), the association between maternal smoking around birth and childhood asthma was still statistically significant (*P* < 0.05) (see Figures 2–4; Table 4).

**Table 4:** Multivariable Mendelian randomization results of maternal smoking around birth on childhood asthma.

**Exposure index**	**OR(95%CI)**	**P**
Maternal history of Alzheimer's disease	0.9997 (0.9991–1.0004)	0.4510
Maternal smoking around birth	1.0146 (1.0026–1.0267)	0.0170
Maternal smoking around birth	1.0134 (1.0012–1.0257)	0.0307
Illnesses of the mother: high blood pressure	0.9972 (0.9877–1.0068)	0.5704
Illnesses of the mother: heart disease	1.0001 (0.9835–1.0170)	0.9908
Maternal smoking around birth	1.0148 (1.0024–1.0274)	0.0188
Maternal history of Alzheimer's disease	0.9997 (0.9990–1.0003)	0.3311
Maternal smoking around birth	1.0128 (1.0008–1.0250)	0.0361
Illnesses of the mother: high blood pressure	0.9962 (0.9865–1.0060)	0.4410
Maternal history of Alzheimer's disease	0.9997 (0.9991–1.0004)	0.4102
Illnesses of the mother: heart disease	0.9987 (0.9825–1.0151)	0.8730
Maternal smoking around birth	1.0146 (1.0024–1.0269)	0.0184
Illnesses of the mother: heart disease	1.0026 (0.9857–1.0199)	0.7633
Maternal smoking around birth	1.0130 (1.0006–1.0254)	0.0397
Illnesses of the mother: high blood pressure	0.9964 (0.9860–1.0070)	0.5063
Maternal history of Alzheimer's disease	0.9996 (0.9990–1.0003)	0.3011
Illnesses of the mother: heart disease	1.0011 (0.9844–1.0182)	0.8946
Maternal smoking around birth	1.0126 (1.0003–1.0249)	0.0438
Illnesses of the mother: high blood pressure	0.9957 (0.9851–1.0064)	0.4263

## Discussion

**Figure 5:**
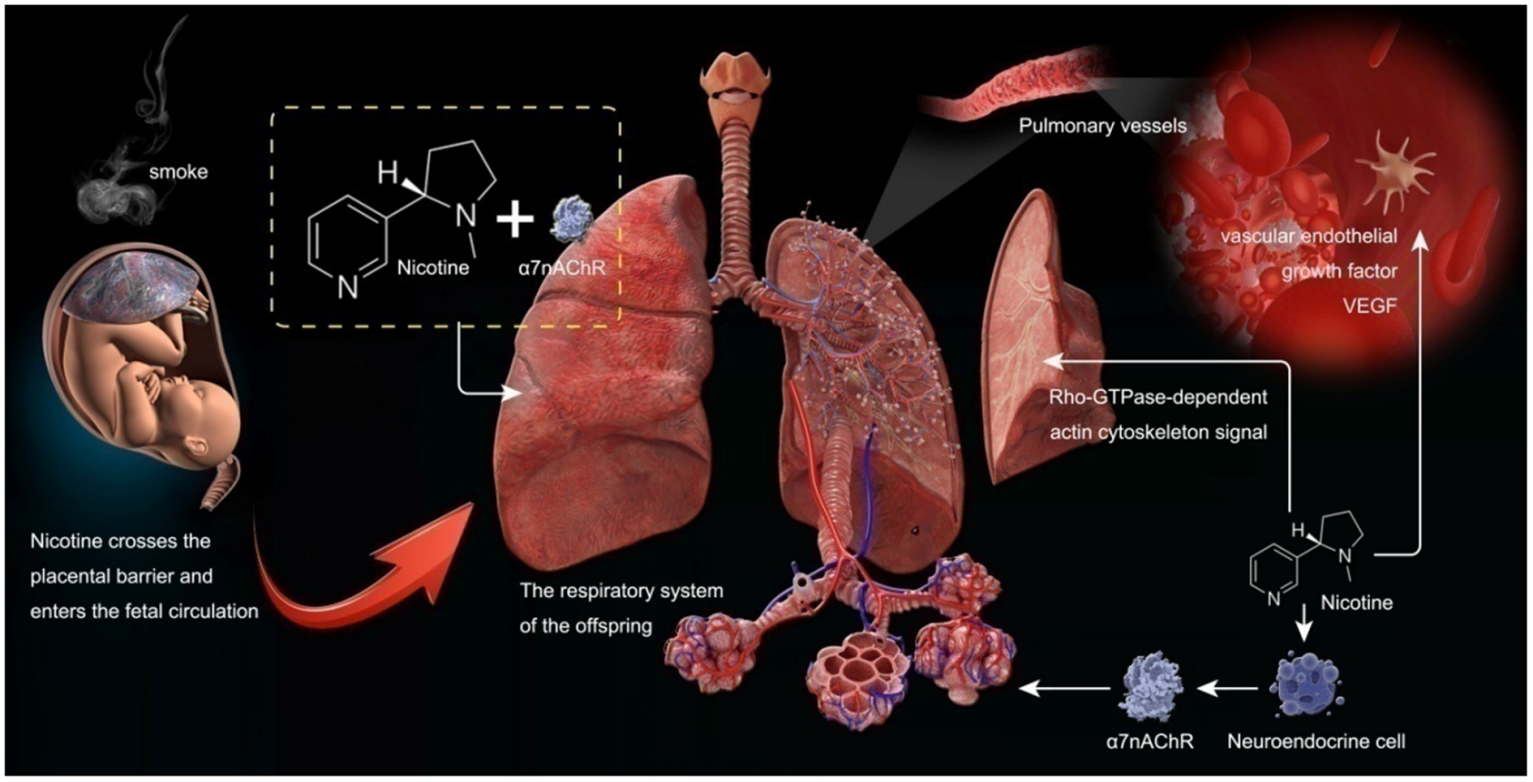
The pathological mechanism of maternal smoking around birth on childhood asthma.

Pregnant women who smoke expose their babies in the uterus to smoke. This study used the MR method to confirm the causal relationship between maternal smoking and childhood asthma. Maternal smoking can affect the lung condition of offspring and lead to asthma, according to the discussions below (see Figure 5).

Smoking in pregnant women can affect the lung development of their offspring. Tobacco smoke contains thousands of chemical compounds. In recent years, numerous studies have shown that smoking during pregnancy and smoking stimulation can affect the lung development of offspring. Nicotine, as one of the leading chemical components in smoke, can enter fetal circulation through the placental barrier and interact with nicotinic acetylcholine receptors (nAChRs) in the fetal lung. This interaction may lead to changes in the structure and function of the lung of offspring. Nicotine can also induce the proliferation of fetal lung neuroendocrine cells, stimulate the expression of nAChRs, and affect the development of the lung of offspring. NAChRs are composed of five transmembrane subunits (8). Evidence hasshown that cigarette smoke stimulation during pregnancy can increase the expression of α7 nicotinic acetylcholine receptor (α7nAChR) in fetal lungs, airway cartilage, and blood vessels (9). A recent study on lung tissues of fetal and COT death cases also found that the expression of α7nAChR in the lungs of offspring whose mothers smoked was significantly increased and positively correlated with fetal lung hypoplasia (10). This is consistent with animal studies in which prenatal nicotine exposure in mice reduced forced expiratory flow in adulthood but not in α7nACachR knockout mice (11). Such results have proven that smoking during pregnancy can stimulate the increase of α7nACachR expression in the respiratory system of offspring and affect the lung development of offspring.

Long term smoking during pregnancy can lead to the accumulation of collagen on the surface of fetal lung parenchyma, increase the expression of pulmonary surfactant protein gene, and induce the proliferation of fetal pulmonary neuroendocrine cells (12), thus producing acetylcholine. In addition, long-term smoking during pregnancy may also promote the synthesis of acetylcholine in fetal lung tissue, and acetylcholine is a component of cholinergic autocrine ring, which is one of the key processes of fetal lung development.

Proskocil et al. (13) found that prenatal exposure to nicotine in pregnant women may affect lung development by changing the autocrine cholinergic cycle. Angiogenesis is essential for alveolarization during normal lung development, and vascular endothelial growth factor (VEGF) is a heparin-binding growth factor specific to vascular endothelial cells, potentiallyinducing neovascularization *in vivo*. VEGF plays a critical role in lung development. Numerous studies have shown that cigarette smoke stimulation can affect the expression of VEGF (14). Jiang et al. (15) showed that long-term exposure to nicotine during long-term pregnancy could reduce the expression of vascular VEGF in offspring rats and further affect the development of lung structure in offspring rats. Petre et al. (16) also found that cigarette smoke exposure during pregnancy reduced pulmonary vascular endothelial growth factor in offspring, thus affecting lung development. These findings suggest that maternal cigarette smoke exposure during pregnancy can reduce the expression of VEGF in the lung vessels of offspring and affect the lung development of offspring.

Pulmonary dysplasia and childhood asthma showed some similar symptoms and signs. Been et al. (17) conducted a meta-analysis of 1543639 children involved in 30 studies. It showed that premature delivery would increase the risk of asthma, and pulmonary dysplasia was also the most common complication of premature delivery.

One common feature of pulmonary dysplasia and asthma is that the patients with these two diseases are in a low antioxidant state. The oxygen free radicals generated by the body stimulated by oxidative stress cause various forms of damage to cells and molecules. Intervention with anti oxidant drugs can effectively prevent and antagonize the damage caused by oxygen free radicals. Asthmatic children often have vitamin A deficiency (18); Vitamin A deficiency will lead to pulmonary dysplasia of premature infants; On the contrary, vitamin A supplementation will alleviate the demand of premature infants for oxygen support. Vitamin A can antagonize the oxidative injury of respiratory tract, promote the repair of injury, induce the immune tolerance of the body, and inhibit the high reactivity of respiratory tract. The common feature of pulmonary dysplasia and asthma is the response of bronchial smooth muscle. Growth transformation factor β (TGF-β). And extracellular matrix are highly expressed in children with asthma (19) and pulmonary dysplasia, and both of them have airway hyperresponsiveness, which can also prove the correlation between asthma and pulmonary dysplasia (20).

Cigarette smoke exposure can also lead to the proliferation of interstitial lung tissue in offspring, affecting lung growth and development. Unachukwu et al. (21) found that the offspring of pregnant mice exposed to cigarette smoke had reduced lung volume in their offspring mice, which may be related to the loss of interstitial lung tissue and decreased cell proliferation. Moreover, they also found that smoke could induce the upregulation of the Rho-GTPase-dependent actin cytoskeleton signal through lung tissue gene detection. It can lead to the loss of lung tissue integrity in offspring. England et al. (22) also found, in non-human primates, that cigarette smoke exposure during pregnancy reduced the size and volume of the lungs of the offspring of the primates. These findings further suggest that maternal smoking during pregnancy can affect the lung development of offspring.

Cigarette smoke by oxidation-antioxidant imbalance leads to oxidative stress, and its role in the development of lung disease is now clear. In addition, it has been shown in previous studies that pregnant women who smoked increased the oxidative stress level in the fetus, reduced the antioxidant capacity of the lungs, and promoted pulmonary fibrosisand premature pulmonary aging of their babies (23). Guzel (24) found that prenatal exposure to cigarette smoke-exposed puppies increased lung oxidative stress levels and oxidative lung damage, thickened the alveolar interval, caused partial degeneration of the tracheal and bronchial lumen of cellular debris, and transparent membrane formation. Still, this result was not observed in their group of antioxidant animals.

Smoking during pregnancy can affect the lung function of the offspring. It is well-known that cigarette smoke stimulation is one of the primary causes of lung disease. Many studies have shown that long-term cigarette smoke exposure during pregnancy can affect the growth of offspring lung structure, mainly manifested in one aspect influencing fetal alveolar mineralization to reduce the number of alveoli. On the other hand, it can also cause fetal alveolar-bronchial attachment reduction, leading to airway stenosis. The above two points have specific effects on lung function. It has been shown in some studies that the decrease in pulmonary function in children may be related to structural changes in the fetal airway/lung (23). In a recent birth cohort study, the offspring of women who smoked during pregnancy had significantly increased airway resistance and decreased forced expiratory volume in 1 s. Their FEV1/forced vital capacity (FVC) ratio was reduced (25).

Several birth cohort studies of longitudinal pulmonary function tests have shown that maternal smoking during pregnancy is associated with sustained impairment of expiratory flow in the offspring. Moshammer et al. (26) studied more than 20,000 children aged 6–12 years in Europe and North America. These researchers found that intrauterine smoke exposure was associated with decreased lung function in offspring, with a 4% decrease in medium-term mean expiratory flow and a 40% increase in the risk of poorer lung function (27). In a recent 21-year follow-up prospective study, the male offspring of mothers who smoked during pregnancy were found to have a continued decrease in their FEV1 and FEF (25-75) (forced expiratory flow between 25 and 75% of FVC) (27). Shagiwal et al. (28) studied 3,347 10-year-old children. They found that adult-related genetic variations in lung function were associated with offspring lung function, and maternal smoking during pregnancy further reduced FEV1, FEV1/FVC, and FEF75 in children. Stocks et al. (29) also found that at birth and before any significant exposure to postpartum smoke, infants born from smoking mothers showed decreased lung function, decreased respiratory flow and respiratory compliance, and altered tidal breathing patterns. These results further support the effect of intrauterine smoke exposure on fetal lung development. Similar results were also obtained in related animal studies. For example, Drummond et al. (30) found that the lung function of mice exposed to cigarette smoke before birth was significantly reduced. Spindel et al. (31) also found reduced lung compliance and forced expiratory flow in mice exposed to prenatal nicotine. Additionally, Joubert et al. (32) found that maternal smoking during pregnancy can also lead to the methylation of offspring. For example, bone morphogenetic proteins (BMPs) are essential in linking maternal smoking with reduced lung function in offspring. All these findings prove that cigarette smoke stimulation during pregnancy can affect the development of offspring lung function.

Smoking during pregnancy can increase the incidence of asthma in offspring. Maternal smoking during pregnancy can affect the development of lung structure and lung function of offspring, thus significantly increasing the incidence of asthma in these individuals.

Increased risk of wheezing and asthma in children: Wheezing and asthma are the most common chronic health problems in childhood, and maternal smoking smoke stimulation during pregnancy is the leading risk factor (33). Galobardes et al. (34) showed that cigarette smoke exposure was a significant risk factor for asthma. In a birth cohort study of 18,041 children, Hallit et al. (35) found that maternal smoking was a risk factor for persistent or intermittent asthma attacks in offspring within the first year of life. A higher incidence of asthma in the offspring of mothers who smoked during pregnancy has also been found in other birth cohort studies (36). Den et al. (37) investigated 6,007 children and found that mothers who smoked continuously during pregnancy had an increased risk of early and persistent wheezing and asthma in their children. Additionally, smoking in pregnant women was reported in a recent meta-analysis to have significantly increased the risk of asthma in children of all age groups (38).

It has been clinically confirmed that maternal smoking leads to an increased risk of asthma in offspring. However, the specific mechanism is unclear. An animal study showed that maternal smoke exposure during pregnancy could increase airway remodeling, allergic airway inflammation, and airway hyperresponsiveness in offspring (39). Southam et al. (40) showed that acetylcholine reactivity was associated with airway smooth muscle thickening. However, in a study by Elliot et al. (41), increased acetylcholine chloride reactivity in offspring from smoke-exposed guinea pigs was not associated with an increased smooth muscle area. This may be related to their finding that there was no significant change in smooth muscle layer thickness 21 days after birth in guinea pigs. Additionally, Kabesch et al. (42) used a birth cohort study to find that c-Jun N-terminal kinase 2 (JNK2) hypomethylation in offspring born from smoke exposure *in utero* could increase the risk of late-onset asthma by 40%. Fetal lungs are susceptible to intrauterine smoke exposure, and nicotine has been shown to cross the placenta and interact with the nicotinic acetylcholine receptors expressed in fetal lungs. Nicotine causes lasting changes in fetal lung function after birth, as evidenced by decreased forced expiratory flow during later pulmonary function measurements. Reduced lung function can lead to wheezing, asthma, and an increased risk of respiratory infections in children.

## Conclusion

In conclusion, this study used genetic data to explore the causal relationship between maternal smoking and maternal diseases and asthma in offspring. The results showed that maternal smoking increased the risk of asthma in offspring, suggesting that maternal non-smoking can prevent asthma in offspring. Good pregnancy habits should be taken seriously throughout the whole pregnancy.

## Data availability statement

The datasets presented in this study can be found in online repositories. The names of the repository/repositories and accession number(s) can be found in the article/supplementary material.

## Ethics statement

The authors are accountable for all aspects of the work in ensuring that questions related to the accuracy or integrity of any part of the work are appropriately investigated and resolved. The study doesn't include human and animal experiments.

## Author contributions

ZD, LP, HC, FL, and MW was responsible for the study design, got data from GWAS, completed the data analysis, and drafted the manuscript. All authors have read and approved the final manuscript.

## Funding

This study was supported by the scientific research project of Shanxi Provincial Health Commission (No. 2022073).

## Conflict of interest

The authors declare that the research was conducted in the absence of any commercial or financial relationships that could be construed as a potential conflict of interest.

## Publisher's note

All claims expressed in this article are solely those of the authors and do not necessarily represent those of their affiliated organizations, or those of the publisher, the editors and the reviewers. Any product that may be evaluated in this article, or claim that may be made by its manufacturer, is not guaranteed or endorsed by the publisher.
